# Examining physiological parameters of mental workload in the cockpit: a multimethod approach

**DOI:** 10.3389/fphys.2026.1893570

**Published:** 2026-07-23

**Authors:** Anneke Hamann, Raphael J. Kneffel, David Cyrol, Elena Rankova, Stefan Sammito, Nils Carstengerdes

**Affiliations:** 1 Deutsches Zentrum für Luft- und Raumfahrt e.V. (DLR), Institut für Flugführung, Braunschweig, Germany; 2 German Air Force Centre of Aerospace Medicine, Research & Development, Cologne, Germany; 3 Department of Occupational Medicine, Faculty of Medicine, Otto von Guericke University Magdeburg, Magdeburg, Germany

**Keywords:** aviation, mental workload, multimethod, multimodal, physiology, pilots

## Abstract

With increasing automation in the cockpit and the focus on adaptive assistance systems that provide tailored assistance to the pilots, it becomes increasingly relevant to monitor the pilots’ state. While many different psychophysiological measurement methods have been utilized in previous studies, they each have drawbacks and limitations that need to be overcome to provide a valid and reliable operator state assessment. In the present study, we investigated the assessment of mental workload (MWL) using a multimethod approach and combination of different physiological measurements (including neurophysiological, central and peripheral measures as well as eye-tracking) during a simulated flight task. A 60-minute simplified flight task was conducted with 14 commercial airline pilots in four difficulty levels, while their physiological, performance and self-report data were collected. Our analyses reveal that especially neurophysiological measures (electroencephalography [EEG], functional near-infrared spectroscopy [fNIRS]) and measures of heart rate (HR) and heart rate variability (HRV, specifically avNN) were able to differentiate the induced levels of MWL, and that different measures performed better in low and others in high MWL levels. Thus, we argue for a combination of physiological measures to assess MWL for future cockpit applications.

## 1 Introduction

Piloting an aircraft is a demanding activity that requires high cognitive and psychomotor skills as well as profound knowledge about the aircraft system (Stokes and Kite, 1997). Traditionally, the pilots’ tasks fall into the four categories: aviate (controlling the aircraft), navigate (locating one’s position and following the planned route), communicate (radio telephony with air traffic control or other relevant stations) and manage systems (monitoring the aircraft status) (Schutte and Trujillo, 1996). Over the years, aircraft have become more automated. Modern aircraft can fly and land mostly automatic and include advanced onboard assistance systems that compile and analyze aircraft status data and provide calculations of fuel consumption or optimal trajectories (Billings, 1991; Bilimoria et al., 2014). This trend in automation may not shift the priority of the pilots’ tasks, but it changes the proportions of the tasks the pilots have to do themselves. In addition, with increasingly sophisticated and interacting on-board assistance systems, monitoring and managing the automation adds new complexity, requires profound knowledge about the assistance systems and can thus increase the pilots’ mental demand (Sheridan, 1999; 2012; Bainbridge, 1983).

This is not only the case in civil airliners. Military aircraft have seen an increase in automation as well (Shklarsky and Shamir, 2023), expanding the pilots’ roles beyond the control of their own aircraft. Pilots of modern high-performance aircraft, particularly 5^th^ generation and future 6^th^ generation fighter aircraft, are increasingly required to act as mission managers. Their tasks extend far beyond manual flight control and include the integration, prioritization and interpretation of large amounts of sensor data, communication and tactical information under time-critical conditions (Smith and Schnell, 2026; Stensrud et al., 2024). This shift suggests that mental workload (MWL) will increase substantially, especially in operational contexts characterized by uncertainty, high speed and simultaneous task demands.

MWL is a central concept in aviation research and other safety-critical domains, and has been focus of research activities since the 1960s (Gartner and Murphy, 1976; Dehais et al., 2020). While there have been many attempts to classify, characterize and define MWL (Dehais et al., 2020; Martins, 2016), it can generally be defined as the part of an operator’s limited cognitive resources that is needed to perform a given task (O'Donnell and Eggemeier, 1986). The more difficult or demanding the task, the more cognitive resources the operator needs to assign to it in order to keep an adequate task performance. Once the task demands exceed the remaining cognitive resources, task performance will decline and the operator will experience a subjective feeling of overload (O'Donnell and Eggemeier, 1986). On the other hand, too easy tasks may lead to underload and task disengagement, which can eventually also lead to errors and prolonged reaction times (Young et al., 2015).

Ironically, the shift from physical to cognitive tasks that comes with increasing automation prompts the development of yet another layer of assistance systems, this time to help balance the pilots’ MWL to ensure flight safety and optimize mission effectiveness, e.g. by adapting the information density or abstraction level to their needs (Huttner et al., 2024). This kind of tailored, adaptive assistance, however, needs information about the pilots’ state and level of MWL, preferably in real-time, continuous, non-intrusive and as objective, valid and reliable as possible (Hinss et al., 2022; Scerbo, 1996). For this purpose, researchers have looked into physiological and behavioral measurements (Boumann et al., 2023; Borghini et al., 2014; Charles and Nixon, 2019; van Weelden et al., 2022; Young et al., 2015; Pongsakornsathien et al., 2019; Shaw and Harrell, 2023). There is a large and ever-growing body on research on the measurement of central and peripheral physiological parameters like heart rate/heart rate variability, respiration, body temperature or skin conductance (Da Tao et al., 2019; Wang et al., 2024), brain activity (Causse et al., 2015; Gateau et al., 2018), and eye movement (Di Nocera et al., 2007; Faulhaber et al., 2022), among others, and their relation to MWL. The studies vary in fidelity, from layperson samples (Hamann and Carstengerdes, 2022) to experts (Causse et al., 2019; van Weelden et al., 2025), from computer experiments (Herff et al., 2014) to flight simulators (Verdière et al., 2018) and real aircraft (Dehais et al., 2019; Dussault et al., 2004), and in the combination of sensors.

Since it is important to find measures that are not just sensitive to changes in MWL, but also specific to it (Matthews et al., 2015; Hamann and Carstengerdes, 2023), combining and analyzing several psychophysiological parameters at the same time may provide a better picture of the pilots’ MWL and the body’s reactions to the load (Vanneste et al., 2021). Thus, in many studies, researchers use a combination of two or three measurement methods (Bosurgi et al., 2025; Rivas-Vidal et al., 2025; Roques et al., 2025). In rare cases, up to five different measures are used (Matthews et al., 2015; Hristov et al., 2026; Eniyandunmo et al., 2025). More recent papers also utilize machine learning and data fusion techniques to improve the measurement quality, albeit with varying degrees of success (Rivas-Vidal et al., 2025; Salam et al., 2026; Eniyandunmo et al., 2025; Liu et al., 2025; Roques et al., 2025). Yet, between all the study setups, measurement and data analysis methods, to this date there is still no combination of sensors that clearly outperform others and provide a precise MWL measurement. Perhaps the broadness and heterogeneity of the body of research is not just a strength but a weakness because it makes it difficult to generalize findings on certain measurement methods beyond the used experimental paradigm and sample. Moreover, the higher the fidelity of a study, the less control of covariates such as mental fatigue or sleepiness is possible (Sammito et al., 2022), further complicating the generalizability of findings between studies.

In the present research, we wanted to overcome these problems by inducing different levels of MWL in pilots in a controlled experimental setup and measuring the associated physiological and behavioral changes with a large, heterogeneous multimodal set of eight concurrent measurement methods, namely electroencephalography (EEG), functional near-infrared spectroscopy (fNIRS), electrocardiography (ECG), blood pressure, respiratory rate, skin conductance, peripheral skin temperature and eye-tracking. We conducted our study in a high-fidelity flight simulator and recruited commercial airline pilots to obtain ecological validity. At the same time, we utilized a simplified flight task/working memory paradigm for manipulating MWL by means of task difficulty while controlling for mental fatigue in a one-factorial (task difficulty) within-subject design that had proved successful in past research. We were primarily interested in analyzing which of the collected measures were sensitive to changes in MWL and could differentiate multiple levels of task difficulty. Specifically, we hypothesized that increasing task difficulty would result in increasing cognitive demand and associated higher self-reported MWL, lower task performance, changes in physiological and behavioral responses. This way, we hoped to get a clear and broad picture of the utility of multiple measures for assessing changes in MWL, and be able to draw conclusions on the optimal combination of measurement methods for assessing MWL in a cockpit environment. This study has been pre-registered at AsPredicted.org: https://aspredicted.org/c6r2-wjjv.pdf.

## 2 Materials and methods

### 2.1 Sample

For this study, we recruited 16 active, licensed commercial pilots with an Airbus A320 family type rating. Recruitment was done between September 2023 and February 2024. Further inclusion criteria were: right-handedness, native German speakers, normal hearing and normal or corrected-to-normal vision. The participants were informed verbally and with written information, provided written informed consent and received monetary compensation for participation. All participants were instructed to follow their usual sleep and caffeine habits prior to the study, and were screened for sleepiness prior to the experiment using the Karolinska Sleepiness Scale (KSS; Akerstedt and Gillberg, 1990; German translation by Niederl, 2007). The KSS is rated on a scale from 1 (“extremely alert”) to 9 (“very sleepy”). If participants indicated a 7 (“sleepy but no difficulty remaining awake”) or higher, their data were excluded from the analysis, which applied to two participants. The final sample thus consists of 14 pilots. All were male, aged 27 to 49 years (*M* = 35.6, *SD* = 6.0), with flight experience between 950 and 17,500 hours (*M* = 4,039, *SD* = 4,363). One participant indicated vision impairment in the questionnaire, but had no trouble during the experiment, and was thus not excluded from the final sample. The study was approved by the medical ethics commission of the North Rhine State Chamber of Physicians, Germany (No. 2023190), and conducted in accordance with the Declaration of Helsinki.

### 2.2 Flight simulation and experimental task

The study was conducted in the flight simulator AVES of the Institute of Flight Systems at the German Aerospace Center (DLR) Braunschweig, a high-fidelity A320 cockpit simulator. The flight task was simplified and designed for one participant, analogous to the task used by Hamann and Carstengerdes (2022). Only cruise flight was simulated, with good weather conditions and nominal aircraft functions. All participants sat on the right seat in the cockpit (First Officer). They were instructed to only use the primary flight display in front of them as well as the heading dial and vertical speed dial. All other functions were controlled by the autopilot.

The flight simulation lasted approx. 60 minutes and consisted of 8 task blocks of about 5 minutes each. During each block, the participants performed two parallel cognitive tasks, an adapted n-back task and a monitoring task (Hamann and Carstengerdes, 2022). In the monitoring task, the participants had to monitor and maintain the altitude of the aircraft, initially set to 20,000 ft. Deviations from this altitude were triggered by the experimenter, and if the deviation exceeded 40 ft in either direction, the participants had to correct back to the initial altitude. In the adapted n-back task, working memory load was manipulated in four levels (0- to 3-back). Participants had to set the heading of the aircraft according to auditory heading commands and the respective n-back level. For instance, in a 1-back condition, the participants had to memorize one heading command each, and put in the heading one prior to the one just heard. The same n-back level was maintained throughout one block, and n-back levels were presented pseudo-randomized, i.e. the same n-back level never appeared twice in a row. During each block, 13 heading commands were given, with an inter-stimulus interval of *M* = 22 s, *SD* = 3 s. More details on the construction of both tasks can be found in Hamann (2023).

### 2.3 Procedure

Upon arrival, the participants were briefed about the experiment, gave written informed consent and filled in a demographic questionnaire. They then proceeded to the simulator, received a short safety briefing, instructions for the tasks and practiced the tasks in parallel. During practice, the difficulty levels of the n-back were presented in ascending order (0- to 3-back). Participants needed to achieve min. 60% correct reactions to move to the next difficulty level. Thereafter, the physiological measurements were placed on the participants. They then rated their momentary sleepiness on the KSS and started the main experiment during which the physiological data were recorded. After each block, participants gave verbal ratings of their subjective MWL (Instantaneous Self-Assessment [ISA]; Tattersall, 1994; Tattersall and Foord, 1996) and mental fatigue (MF; Fatigue Instantaneous Self-Assessment [F-ISA]; Hamann and Carstengerdes, 2020), followed by a blood pressure measurement and a break of approx. 2–3 minutes. After the experiment had finished, participants gave a second KSS rating, were debriefed, thanked and seen off.

### 2.4 Physiological data recording and pre-processing

EEG data acquisition was performed with a LiveAmp-32 device and in BrainVision Recorder 1.25 (Brain Products GmbH, Gilching, Germany) at 500 Hz. Twenty-six Ag/AgCl active EEG electrodes were positioned according to the 10–20 system with online reference at FCz. Six electrodes around the ears were left out to account for the eye-tracking glasses. The data pre-processing was done in BrainVision Analyzer 2.3 (Brain Products GmbH, Gilching, Germany). The raw data were down-sampled to 256 Hz and re-referenced to average. A 4^th^ order IIR bandpass filter between 0.5–40 Hz was applied as well as an additional 50 Hz notch filter to remove remaining excessive line noise from the simulator environment. Ocular artefacts were corrected with an Ocular Correction Independent Component Analysis (Restricted Infomax) on the whole data, with electrode Fp1 for identifying vertical and horizontal eye activity. Components were rejected based on the computed energy values and expert judgment. Further movement and recording artefacts were rejected semi-automatically based on threshold values of gradient, difference, amplitude and low activity per electrode as well as expert judgement. On average, 0.06% of data was excluded. The data were then divided into blocks as described previously, and further segmented into epochs of 2 seconds with 0.5 seconds overlap in a moving-window fashion. Next, the segments were transformed into the frequency domain using the Fast Fourier Transformation with maximum frequency resolution (0.5 Hz). A 10% Hanning window was applied to reduce discontinuity and spectral leakage between segments. Power Spectral Density (µV²/Hz) was computed per segment and averaged for each 5-minute block. The resulting values were exported as raw sum for the theta band (4–8 Hz) at electrodes Fz, F3 and F4, and for the alpha band (8–12 Hz) band at electrodes Pz, P3, P4.

**Figure 1:**
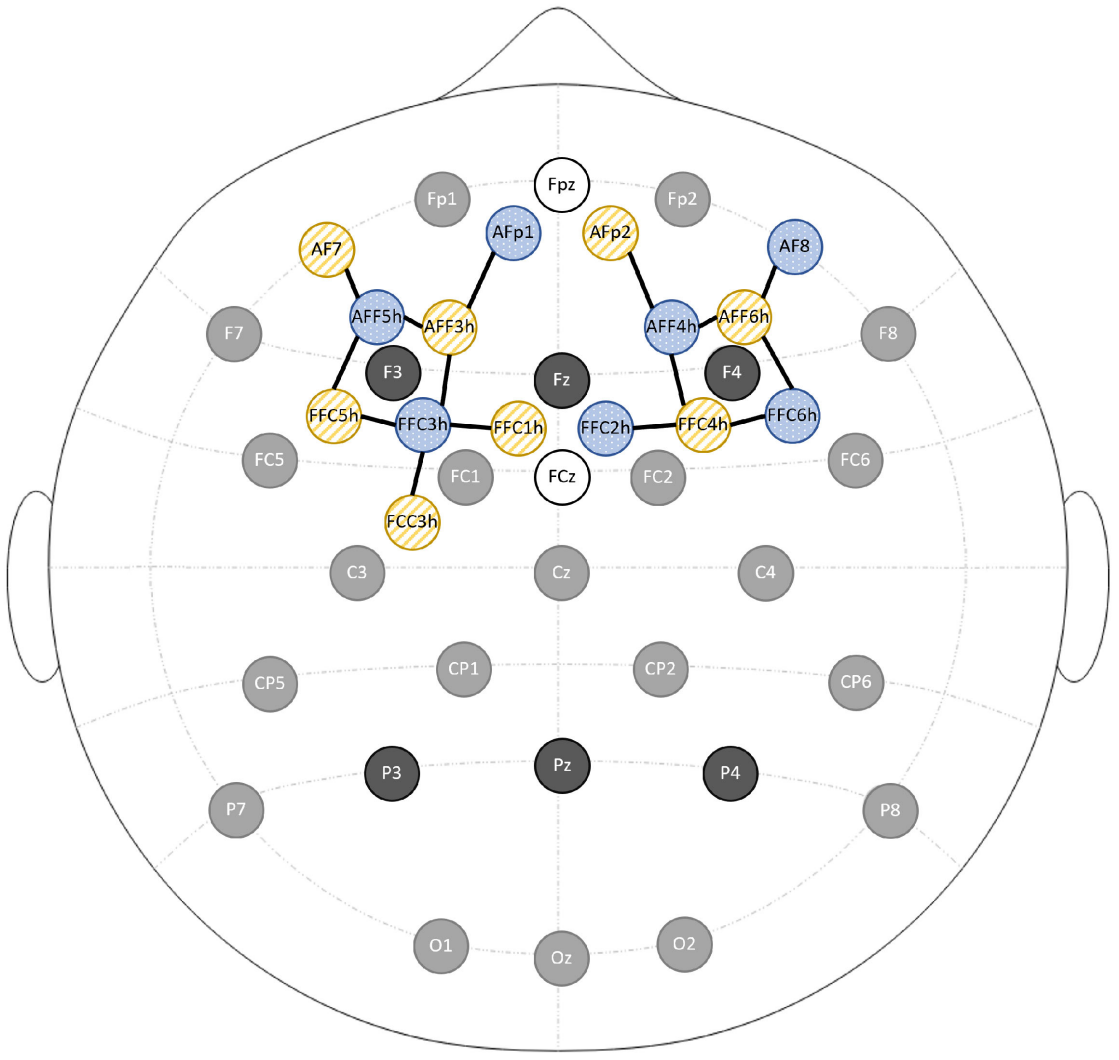
Combined EEG-fNIRS montage. EEG: white = ground and reference electrodes; grey = electrodes (dark grey = electrodes used in the analysis); fNIRS: striped yellow = source incl. short distance channel; dotted blue = detector; black lines = resulting fNIRS channel.

The fNIRS data were recorded with a NIRSport2 device (8 sources, 7 detectors, 8 short-distance channels) and Aurora 2021.9 (NIRx Medical Technologies LLC, Glen Head, NY, USA) at 10 Hz. The optodes were positioned between the EEG electrodes in a custom montage analogous to the one used by Hamann and Carstengerdes (2022), see **Figure 1**. fNIRS pre-processing and analysis were performed with the NIRS Brain AnalyzIR toolbox for MATLAB (Zimeo Morais et al., 2018). The raw voltage data were down-sampled to 4 Hz, converted to optical density transformed into HbO and HbR concentrations using the modified Beer-Lambert Law (Jacques, 2013). The correction of systemic noise, motion artefacts and serial autocorrelation is done via regression and thus described in the analysis section.

**Figure 2:**
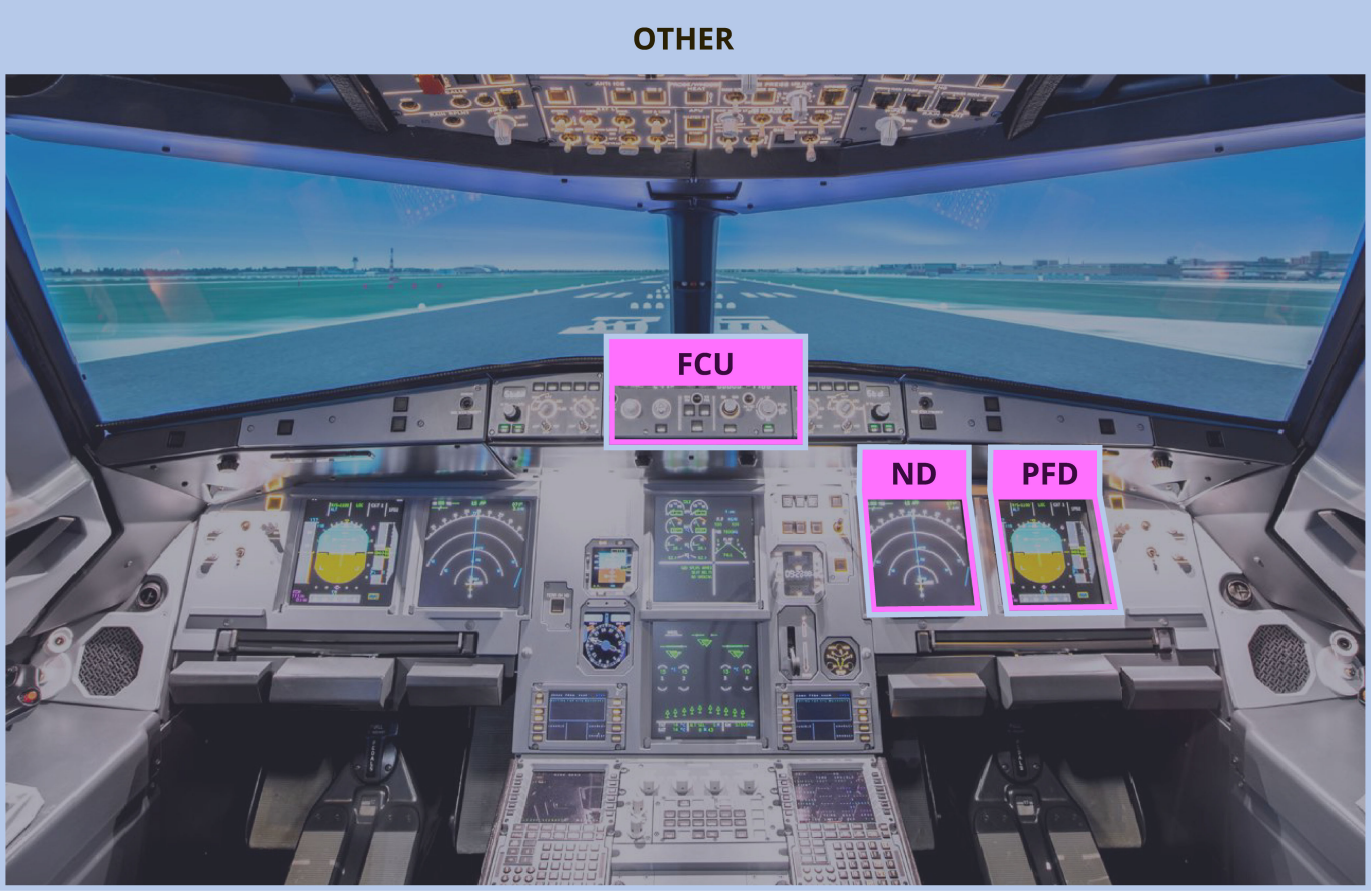
Overview of the simulator cockpit and the AOIs designated for the eye-tracking analysis. Flight Control Unit (FCU), Navigation Display (ND), Primary Flight Display (PFD), and OTHER – an AOI capturing all fixations outside the FCU, ND, and PFD displays. Photo: DLR.

Ocular data were collected at 50 Hz using the wearable Tobii Pro Glasses 3 (Tobii AB, Danderyd, Sweden). This eye-tracker calculates gaze position using the dark pupil Pupil-Center Corneal Reflection (PCCR) method. It operates by illuminating the eyes with infrared light and capturing corneal reflections with four infrared cameras arranged in a stereo geometry. This eye-tracker achieves an accuracy of 0.6° and provides a Field-of-View of about 106° diagonal. For two participants, the eye-tracking data could not be used because the data files were corrupted during the recording. The remaining eye-tracking data were processed using the Tobii Pro Lab software (version 1.241.54219). Fixations were determined using the Tobii I-VT (Attention) gaze filter, a velocity-based filter specifically adjusted for the Tobii Glasses with a threshold value of 100°. For exploratory analyses, four Areas of Interest (AOI) were defined: Flight Control Unit (FCU) which was relevant for the n-back and monitoring tasks (giving input); Primary Flight Display (PFD) which was relevant for the monitoring task; and Navigation Display (ND) which was irrelevant to both tasks and should serve as control. The remainder of the cockpit was defined as “other”, see **Figure 2**. The gaze position in relation to the selected AOIs was determined using the Tobii Snapshot functionality, followed by a manual review and correction of the results. For exploratory analyses, we were interested in changes in the dwell time spent on each AOI and how often participants would shift their gaze between AOIs. Thus, five measures were extracted from the data for each task block: Dwell time percentage (%) per AOI (FCU, PFD, ND), and the transition frequency (in number of transitions) between all areas (the three AOIs and the “other” area), and only between FCU and PFD.

Three-channel-ECG (sampling rate 1000 Hz), respiratory rate (each breathing was measured), blood pressure (once after each 5-min block), skin conductance (sampling rate 10 Hz) and peripheral skin temperature measurement (sampling rate 10 Hz) were recorded via corresponding satellites of the mobPhysioLab (Kora Industrie-Elektronik GmbH, Hambühren, Germany). For this purpose, the HFM-Flash Master was connected to the SAT-02 satellites for the ECG sensors with three electrodes on the chest of the participant (one on the right top right corner, one in the top left corner and one in the lower left part of the abdomen), for peripheral skin temperature measurement and skin conductance a sensor at the left hand, the SAT-03 for the respiratory sensor as a chest strap and the SAT-20.02 for additional blood pressure measurement on the left upper arm. Blood pressure was measured oscillographically using the Mobilo Graph NH 24 ABPM and a blood pressure cuff (size M or L) adapted to the size of the subject’s upper arm. The mobPhysioLab was set up in a standardised manner in advance using the associated Heally Control program (Version 8.1.2, SpaceBit, Eberswalde, Germany) and started manually at the beginning of the experiment for continuous measurement. The data was recorded on the corresponding storage medium in the HFM-Flash Master. For each subject and each n-back-level, the heart rate (from the ECG), respiratory rate, peripheral skin temperature and skin conductance were calculated as average over the respective 5-minute block and this value was used for further calculation. The blood pressure measurement took place at the end of each 5-minute block and was started manually. The recorded ECG was exported as an ECG curve and imported into LabChart Pro (Version v8.1.28, AdInstruments, Dunedin, New Zealand) at a sampling rate of 1000 Hz for the purpose of analysing the heart rate and heart rate variability (avNN, SDNN, RMSSD, pNN50, SDSD).

### 2.5 Data analysis

The statistical analyses were performed in IBM Statistics SPSS 24 and 26 (IBM Corp., Armonk, NY, USA) and NIRS Brain AnalyzIR toolbox (Zimeo Morais et al., 2018) for MATLAB (version R2020a). The physiological data were divided into eight blocks according to the n-back task. Because each block varied slightly in length, all blocks longer than 5 minutes were shortened to 5 minutes, measured from the end of the block backwards. Two participants accidentally did a 2-back instead of 3-back during the first presentation of the 3-back condition; the data of these incorrect blocks were treated as missing values for all MWL-related measures.

#### 2.5.1 General analysis strategy

The chosen significance level for all conducted analyses is α <.05. If not stated otherwise, all analyses were carried out following the following procedure: The data were averaged across both presentations per n-back level, resulting in a one-factorial (n-back levels: 0-, 1-, 2-, 3-back) design. Repeated-measures ANOVAs with *post-hoc* Bonferroni-corrected paired t-tests were computed for each measure. In case of violation of the sphericity assumption in one of the ANOVAs, the Greenhouse-Geisser correction was applied. The tested measures and deviations from this general approach (if any) are reported below.

#### 2.5.2 Subjective and performance data

Subjective MWL ratings (ISA), reaction times in the monitoring task (in seconds), and accuracy in the n-back task (in percent, as ratio of correct responses to all responses and misses) were analysed following the general approach. Sleepiness and MF ratings were treated differently: For subjective MF ratings (F-ISA), a one-factorial (time, i.e. 8 blocks) repeated-measures ANOVA was carried out to analyse effects over time. KSS values before and after the experiment were compared using a paired t-test.

#### 2.5.3 Physiological data

The exported EEG PSD values for the theta band (electrodes Fz, F3, F4) and alpha band (electrodes Pz, P3, P4) were checked for skewness and ln-transformed to reach a normal distribution. Initially, a one-way (n-back level) repeated measures MANOVA per frequency band for all three electrodes and follow-up univariate ANOVAs for each electrode were planned. However, due to the small sample size, the df(error) and thus resulting statistical power of the MANOVA would have been too small to interpret reliably. Thus, per frequency band, just the three univariate ANOVAs, one for each electrode of interest, were computed and Bonferroni-corrected to account for alpha inflation.

The fNIRS data were divided into blocks as previously described, and analysed in a two-level general linear model (GLM) using the gamma hemodynamic response function with the n-back levels as predictors. On the first level (subject), the short-distance channels were included as predictors to control for systemic noise and motion artefacts. On the second level (group), serial autocorrelation was corrected for using a pre-whitening algorithm (AR-IRLS; Barker et al., 2013). The obtained beta coefficients were entered into a mixed-effects model with n-back level as the fixed effect and a random intercept for the subject. Contrasts between the n-back levels were computed with t-tests, and *p*-values were corrected using the false-discovery rate (FDR; Benjamini and Hochberg, 1995). In addition to the single channels, two regions of interest (ROIs) used by Hamann and Carstengerdes (2022) were tested.

The five eye-tracking measures (% dwell time per AOI and transition frequencies) as well as the central and peripheral physiological measures were checked for skewness. Since there were no consistent deviations from a normal distribution in any measure, only in some conditions, and ANOVAs are generally robust against such deviations (Schmider et al., 2010), no transformation was applied. All analyses for these measures followed the general approach.

## 3 Results

### 3.1 Subjective and performance data

Subjective MF increased slightly throughout the experiment. The mean KSS ratings showed a significant increase between pre- and post-experiment, *t*(13) = -7.88, *p* <.001, from 3.36 (*SD* = 1.34) to 5.57 (*SD* = 1.56), indicating medium MF levels at the end of the experiment. F-ISA ratings increased over the course of the experiment (between block 1 and 8), *F*(2.59, 33.66) = 7.40, *p* = .001, η²_p_ = .36. Bonferroni-corrected *post-hoc* pairwise comparisons showed significant differences between block 1 and blocks 7 (*p* = .039) and 8 (*p* = .028), respectively. Mean MF increased from 1.64 (*SD* = .75) in block 1 to 2.93 (*SD* = 1.0) in block 8, indicating only medium MF levels as well.

The self-reported MWL (ISA ratings) increased significantly with task difficulty, *F*(3, 39) = 117.14, *p* <.001, η²_p_ = .90. The ratings increased from very low (*M* = 1.21, *SD* = 0.43) in the 0-back condition to high MWL (*M* = 3.96, *SD* = 0.69) in the 3-back condition. Bonferroni-corrected *post-hoc* pairwise comparisons showed significant differences between all n-back levels, all *p*s <.001, see **Table 1**.

**Table 1:** Bonferroni-corrected *post-hoc* pairwise comparisons for subjective and performance data.

	Comparison	1		2		3	
Measure		Mean Δ (SE)	p	Mean Δ (SE)	p	Mean Δ (SE)	p
ISA	**0 vs.**	-0.61 (0.13)	**.003**	-1.29 (0.15)	**<.001**	-2.75 (0.18)	**<.001**
	**1 vs.**			-0.68 (0.15)	**<.001**	-2.14 (0.15)	**.003**
	**2 vs.**					-1.46 (0.17)	**<.001**
n-back (%)	**0 vs.**	0.29 (0.51)	>.999	4.54 (1.81)	.159	27.20 (5.98)	**.003**
	**1 vs.**			4.25 (1.73)	.171	26.90 (6.00)	**.004**
	**2 vs.**					22.70 (5.64)	**.009**

Significant comparisons are highlighted in bold, significance threshold α <.05. Mean Δ (*SE*) indicates mean difference and standard error between the compared n-back levels. ISA, Instantaneous Self-Assessment.

The two performance measures showed different sensitivity to increasing task difficulty. N-back accuracy was significantly impacted by the increase in task difficulty, *F*(1.16, 15.08) = 18.34, *p* <.001, η²_p_ = .59. This effect was mainly driven by the most difficult condition, the 3-back level. Bonferroni-corrected *post-hoc* comparisons showed significant differences only between the 3-back and all other levels, all *p*s ≤.009, see **Table 1**. For monitoring reaction times, there was no effect of n-back level on reaction time, *p* = .145.

### 3.2 Physiological data

In EEG, frontal theta activity could be used to discriminate MWL levels, while parietal alpha activity did not vary substantially with MWL. Parietal alpha activity did not vary significantly at any of the electrodes Pz, P3, P4, all *p*s >.05. Frontal theta activity at the electrodes Fz and F3 increased significantly with increasing n-back level: Fz, *F*(3, 39) = 13.81, *p* <.001, η²_p_ = .52; and F3, *F*(3, 39) = 5.12, *p* = .004, η²_p_ = .28. The ANOVA for electrode F4 did not yield significant results, *p* = .064. *Post-hoc* Bonferroni-corrected comparisons for Fz and F3 can be found in **Table 2**.

**Table 2:** Bonferroni-corrected *post-hoc* pairwise comparisons for EEG theta activity at Fz and F3.

	Comparison	1		2		3	
Measure		Mean Δ (SE)	p	Mean Δ (SE)	p	Mean Δ (SE)	p
Fz	**0 vs.**	-0.05 (0.03)	.574	-0.11 (0.03)	**.011**	-0.18 (0.04)	**.004**
	**1 vs.**			-0.06 (0.02)	.161	-0.13 (0.03)	**.004**
	**2 vs.**					-0.07 (0.02)	.080
F3	**0 vs.**	-0.04 (0.03)	>.999	-0.06 (0.03)	.431	-0.10 (0.03)	**.030**
	**1 vs.**			-0.02 (0.02)	>.999	-0.06 (0.02)	.123
	**2 vs.**					-0.04 (0.02)	.362

Significant comparisons are highlighted in bold, significance threshold α <.05. Mean difference values are Power Spectral Density in µV²/Hz, ln-transformed. Mean Δ (*SE*) indicates mean difference and standard error between the compared n-back levels.

In fNIRS, the left dlPFC was more sensitive to changes in MWL than the right, and HbR was more sensitive than HbO. With HbR, in the left ROI all n-back levels but the two lowest (0- vs. 1-back) could be discriminated, while in the right ROI, discrimination was only possible between the most difficult (3-back) condition and all other conditions, see **Table 3**. In HbO, the results were less consistent across the ROIs, see **Table 3**. Figures depicting these effects can be found in the supplements (**Supplementary Figure 1**, **S2**). The channel-wise results can be found in the supplements (**Supplementary Table 1**).

**Table 3:** Haemodynamic results for ROI t-contrasts. .

	Comparison	1			2			3		
Measure		β (SE)	t	p	β (SE)	t	p	β (SE)	t	p
HbR, ROI left	**0 vs.**	-1.70 (0.94)	-1.82	.100	-4.33 (0.90)	-4.79	**<.001**	-6.76 (0.95)	-7.10	**<.001**
	**1 vs.**				-2.62 (0.92)	-2.86	**.012**	-5.06 (0.97)	-5.22	**<.001**
	**2 vs.**							-2.44 (0.94)	-2.60	**.021**
HbR, ROI right	**0 vs.**	-1.26 (1.30)	-0.97	.574	-0.36 (1.29)	-0.28	.877	-4.45 (1.32)	-3.38	**.004**
	**1 vs.**				0.90 (1.28)	0.70	.728	-3.19 (1.32)	-2.42	**.038**
	**2 vs.**							-4.09 (1.30)	-3.16	**.006**
HbO, ROI left	**0 vs.**	2.62 (2.09)	1.26	.233	-1.87 (2.00)	-0.94	.353	5.86 (2.05)	2.86	**.012**
	**1 vs.**				-4.50 (2.02)	-2.22	**.046**	3.23 (2.11)	1.53	.157
	**2 vs.**							7.73 (1.96)	3.95	**.001**
HbO, ROI right	**0 vs.**	-14.97 (3.04)	-4.93	**<.001**	1.16 (3.01)	0.39	.877	0.68 (3.11)	0.22	.877
	**1 vs.**				16.13 (3.01)	5.36	<.001	15.66 (3.14)	4.98	**<.001**
	**2 vs.**							-0.48 (3.08)	-0.16	.877

*N* = 14, *df* = 52, β = coefficient of the regression model, *p*-values FDR-corrected. Significant comparisons are highlighted in bold, significance threshold α <.05.

For eye-tracking, there were no significant effect of n-back level in any of the measures (transitions between all AOIs, transition frequency between PFD and FCU, percentage of dwell times per AOI), all *p*s >.05.

Of the central and peripheral physiological measures, only some showed significant effects of n-back level. There was a significant effect on heart rate (HR), *F*(1.33, 17.32) = 11.78, *p* = .002, η²_p_ = .46; skin conductance (SRL), *F*(3, 39) = 3.73, *p* = .019, η²_p_ = .22; systolic blood pressure, *F*(3, 39) = 3.17, *p* = .036, η²_p_ = .21; breathing rate (BR), *F*(3, 39) = 5.26, *p* = .004, η²_p_ = .29; and on two of the HRV parameters, avNN, *F*(1.71, 22.17) = 17.09, *p* <.001, η²_p_ = .57; and pNN50, *F*(1.43, 18.57) = 4.57, *p* = .034, η²_p_ = .26. For those, *post-hoc* tests were computed, see **Table 4**. Diastolic blood pressure, peripheral skin temperature and the HRV measures RMSSD, SDNN and SDSS were insensitive to changes in n-back level, all *p*s >.05.

**Table 4:** Bonferroni-corrected *post-hoc* pairwise comparisons for heart rate (HR), breathing rate (BR), skin conductivity (SRL) and blood pressure.

	Comparison	1		2		3	
Measure		Mean Δ (SE)	p	Mean Δ (SE)	p	Mean Δ (SE)	p
HR	**0 vs.**	0.05 (0.67)	>.999	-3.72 (1.14)	**.036**	-8.15 (2.40)	**.021**
	**1 vs.**			-3.77 (1.16)	**.037**	-8.56 (2.29)	**.014**
	**2 vs.**					-4.79 (1.64)	.070
SRL	**0 vs.**	-5.04 (9.00)	>.999	11.02 (9.70)	>.999	19.84 (6.30)	**.046**
	**1 vs.**			16.06 (8.71)	.529	24.89 (8.20)	.057
	**2 vs.**					8.83 (6.59)	>.999
Blood pressure, systolic	**0 vs.**	-0.08 (1.95)	>.999	-1.04 (1.86)	>.999	-5.23 (1.96)	.124
	**1 vs.**			-0.96 (1.41)	>.999	-5.15 (2.11)	.185
	**2 vs.**					-4.19 (2.37)	.610
BR	**0 vs.**	0.19 (0.23)	>.999	-0.23 (.026)	>.999	-1.12 (0.44)	.144
	**1 vs.**			-0.42 (0.33)	>.999	-1.31 (0.40)	**.034**
	**2 vs.**					-0.89 (0.43)	.356
avNN	**0 vs.**	-2.92 (5.08)	>.999	34.84 (7.27)	**.002**	63.91 (14.42)	**.004**
	**1 vs.**			37.76 (8.47)	**.004**	66.83 (13.60)	**.002**
	**2 vs.**					29.07 (12.72)	.238
pNN50	**0 vs.**	-0.01 (0.01)	>.999	0.01 (0.01)	>.999	0.05 (0.02)	.359
	**1 vs.**			0.02 (0.01)	.104	0.06 (0.02)	.132
	**2 vs.**					0.04 (0.02)	.435

Significant comparisons are highlighted in bold, significance threshold α <.05. Mean Δ (*SE*) indicates mean difference and standard error between the compared n-back levels.

**Table 5** gives an overview of the different MWL measures used in this study, and their ability to differentiate the four n-back levels.

**Table 5:** Overview of different mental workload level (MWL) measures and their ability to differentiate the four n-back levels.

	n-back comparison					
	0 vs.			1 vs.		2 vs.
Measure	1	2	3	2	3	3
ISA	✓	✓	✓	✓	✓	✓
n-back (% correct)			✓		✓	✓
monitoring reaction times						
EEG: Theta Fz		✓	✓		✓	
EEG: Theta F3			✓			
EEG: Theta F4						
fNIRS: HbR left		✓	✓	✓	✓	✓
fNIRS: HbR right			✓		✓	✓
fNIRS: HbO left			✓	✓		✓
fNIRS: HbO right	✓			✓	✓	
eye-tracking: % dwell time FCU						
eye-tracking: % dwell time PFD						
eye-tracking: % well time ND						
eye-tracking: all transitions						
eye-tracking: transitions FCU-PFD						
HR		✓	✓	✓	✓	
BR					✓	
SRL			✓			
Temperature						
Blood pressure, systolic						
Blood pressure, diastolic						
avNN		✓	✓	✓	✓	
SDNN						
RMSSD						
pNN50						
SDSD						

Statistically significant differences marked with ✓ on grey.

## 4 Discussion

In the present study, our aim was to assess MWL with a large, heterogeneous multimodal set of eight concurrent measurement methods in order to identify which of these are the most sensitive to changes in MWL and can thus be used and combined for an optimal MWL assessment for cockpit applications. In order to achieve this goal, we let commercial aircraft pilots perform a simplified flight task in a high-fidelity flight simulator and manipulated MWL by increasing the task difficulty via an adapted n-back task with four levels (0- to 3-back).

Our results indicate that this manipulation worked. Subjective MWL ratings increased between the four difficulty levels, and performance in n-back levels decreases with increasing task difficulty (although statistically significant differences could only be found between the most difficult level and all others). Moreover, MF did not confound with MWL in any meaningful way. We recorded a slight increase in MF and KSS ratings across the experiment, from an alert to a medium fatigued state, but no excessive MF or sleepiness was induced. The magnitude of the changes aligns with our previous research (Hamann and Carstengerdes, 2022) even though we increased the duration of the present experiment. Thus, we conclude that the MWL manipulation did not fatigue the participants to an extent that would influence their performance or physiological responses. While we did not see an impact of task difficulty on reaction times in the monitoring task, the performance in the n-back task decreased with increasing task difficulty. Especially the 3-back condition elicited a drop in performance, indicative of elevated MWL and in line with the subjective MWL reports. In sum, we consider our MWL induction successful, and can thus interpret the physiological measures.

In this study, we found that theta activity was sensitive to MWL changes, in line with previous research on working memory tasks (Scharinger et al., 2017; Puma et al., 2018; Grissmann et al., 2017). Electrode Fz was best suited to differentiate MWL, especially for the differentiation of high MWL, while F3 could only differentiate the largest difference between 0- and 3-back. Both electrodes displayed large effect sizes. F4 did not yield any significant results. Thus, central midline measurements outperformed lateralized electrode positions, consistent with the results of our previous study (Hamann and Carstengerdes, 2022). We did not find any significant changes in parietal alpha activity, again consistent with our previous study (Hamann and Carstengerdes, 2022) but contrary to other literature (Grissmann et al., 2017; Scharinger et al., 2017; Radüntz, 2017). We believe this to be due to frequent task switching between the n-back and monitoring tasks (Hamann and Carstengerdes, 2022; Puma et al., 2018), which would cast doubt on the utility of parietal alpha measures for MWL detection in cockpit applications where multitasking is an inherent part of a pilot’s task. In sum, frontal theta activity is a promising candidate for MWL detection and differentiation, with large effect sizes and a special sensitivity for high MWL.

The fNIRS data showed the expected effect of increasing cortical oxygenation (increasing HbO, decreasing HbR) with increasing MWL (Causse et al., 2019; Herff et al., 2014; Gateau et al., 2018). We could not pinpoint single channels that would be most useful for MWL detection, but decided to group channels into RoIs instead, in order to create more robust findings. Using HbR, all MWL levels but 1- vs. 2-back could be discriminated, and the results for the RoI on the left hemisphere were clearer and more consistent than for the right. While this is in line with our EEG results, it contrasts with previous findings (Hamann and Carstengerdes, 2022; Geissler et al., 2020). HbO effects were less consistent than HbR, which aligns with our previous study (Hamann and Carstengerdes, 2022), which could be due to higher contamination from systemic noise than HbR (Huppert et al., 2009; Kirilina et al., 2012). In fact, HbO and HbR results were not always negatively correlated, which serves as strong evidence for contaminated data. With our pre-processing pipeline and the added use of short-distance channels, we took great care to eliminate as much noise from the data as possible. It is, however, possible that the time span of 5 min per block allowed for too much variation, or that pollution from the eye-tracking glasses in the infrared spectrum systematically interfered with the HbO measurement. Further analysis on the effects of block length on the fNIRS data and suitable analysis methods would be necessary, but go beyond the scope of this paper. In sum, HbR measurements seem the better choice for this task and analysis, and their ability to differentiate levels of MWL is comparable with that of the EEG measurement.

The exploratory eye-tracking analyses indicate no effects of task difficulty on the gaze patterns, contrary to previous findings (Faulhaber and Friedrich, 2019). The simplified flight tasks had very specific instructions, only very limited displays to keep track of, and the visual components did not vary between the n-back levels. Thus, the task affordances may have had a stronger influence of gazing behaviour than the task difficulty. This may change in more realistic tasks where the pilots need to monitor even more displays and gather more information than in our simplified task (Faulhaber et al., 2022; Lounis et al., 2021).

Of the peripheral measures, only a few showed main effects of task difficulty and even less could differentiate n-back levels in the *post-hoc* tests. Of the two skin-related measures, only SRL could differentiate MWL levels, and only the lowest from the highest difficulty level, in line with previous research (Mehler et al., 2009). Peripheral skin temperature did not vary with MWL. Skin temperature measurements for MWL assessment are seldom done and yield mixed results (Riaz et al., 2021; Basnet and Zahabi, 2025). Since both measures are influenced by ambient temperature (Boumann et al., 2023), they may not be the best choice for application in a cockpit anyway, especially not in military aircraft without temperature control (Ozaki et al., 2005). BR only differentiated low from high MWL. Systolic blood pressure did not vary with MWL at all; diastolic blood pressure could not differentiate specific MWL levels, in line with previous research yielding mixed results (Lee et al., 2007; Veltman and Gaillard, 1998; Riaz et al., 2021).

The heart-rate related measures were generally more sensitive to changes in MWL: HR itself was able to show significant differences between low and high MWL. The same applied to the HRV parameter avNN, which is directly negatively correlated with HR. Both measures also showed very large effect sizes, meaning they are well-suited for detecting changes. pNN50 showed a significant main effect, but could not differentiate any MWL levels in the *post-hoc* test, making it the least sensitive of the heart-rate related parameters with significant effects. While the sensitivity of heart-rate related measures varies between publications (Zhu et al., 2025; Dussault et al., 2005), our findings are largely congruent with effects described in the literature on the relationship between heart rate and HRV parameters with increasing MWL (Wang et al., 2024; Lohani et al., 2019). In sum, the heart-rate related measures outperformed the other peripheral physiological measures. Yet, although the differences were significant, the absolute changes were sometimes small, so that it would be necessary to investigate if the finding can be repeated if physical stresses are added in additional to a high MWL, such as those acting on the pilot in a high-performance aircraft or in a highly agile helicopter.

In sum, from the multiple method used to differentiate MWL, the neurophysiological measures and HR/HRV performed best. Of the EEG measures, theta Fz activity was most sensitive to changes in MWL, and left HbO changes outperformed the other fNIRS measures. HR and avNN performed equally well in discriminating MWL changes. Yet, from the behavioural and subjective data, it can be inferred that our expert sample did only experience medium, not excessive MWL. On the one hand, this shows that our physiological measurement methods were sensitive enough to able to capture even small to medium changes in MWL and will likely be able to differentiate even higher extents of MWL. On the other hand, some of the methods, especially the peripheral measurements, that did not yield any significant results, may still be useful in detecting excessive MWL. Thus, they should not be discarded lightly, but tested in even more challenging settings.

An obvious limitation of our study is the comparably small sample size. Of 16 recruited participants, only 14 could be included in the analysis, and further data were lost due to technical difficulties. Thus, the statistical power and generalizability of the results could be questioned. Given that the behavioural, subjective, EEG and fNIRS results of this expert sample align to a great extent with our previous findings on a layperson sample (Hamann and Carstengerdes, 2022), we are confident that our results are valid. Nevertheless, it is possible that small effects in some of the used measures could not be found due to the limited statistical power. Thus, follow-up studies with larger samples would be necessary to increase the confidence in and generalizability of our results. In addition, in the current study, we explicitly decided against recruiting military pilots with experience in high-performance aircraft. As the experimental setup targeted general aspects of MWL rather than tasks specific to high-performance aircraft, the use of commercial pilots was considered appropriate for the aims of this study. Yet, for a better generalization to the military domain, future studies should include military pilots and corresponding flight simulators.

In the present study, we took care to eliminate influences of sleepiness and MF by excluding participants with too high KSS ratings, and statistically controlling for MF ratings. We could not, however, control for participants’ flight schedules and associated disruptions of circadian rhythm and fluctuations of attention throughout the day. This somewhat limits the interpretation of the results as pure MWL-associated changes without further confounding variables. On the other hand, this loss of some degree of experimental control makes it easier to generalize our findings to real-life applications. Influences of circadian rhythm and shift cycles are expected in real life as well and come naturally with the extension of research from layperson to expert samples.

This shows that it is important to expand our findings to more realistic, more ecologically valid tasks. Even with the utilization of an expert pilot sample, a high-fidelity flight simulator and a carefully constructed flight task, there is a large gap between our controlled simulator study and real conditions in a cockpit. Different task characteristics, sources of noise and interfering cognitive constructs like task switching (Puma et al., 2018), mental fatigue (Hamann and Carstengerdes, 2023) or emotions (Grissmann et al., 2017) may influence the data and change the validity and reliability of the measurements. This gap becomes even larger when comparing cruise-flight tasks with the mission-management demands expected in future 6^th^ generation fighter aircraft. In such contexts, pilots may need to monitor automated systems, coordinate unmanned aerial vehicles, integrate tactical information and make rapid decisions under uncertainty (Smith and Schnell, 2026; Stensrud et al., 2024). In sum, the present study is a first step to show which physiological and behavioural measures are useful for MWL assessment in a controlled setting, but more research is need to validate our findings in more realistic flight simulations and tasks, before applying them to real flights.

## 5 Conclusions

In summary, utilizing a large number of simultaneously measured central, peripheral, neurophysiological and behavioral parameters, our study was able to show which had potential for differentiating several levels of MWL in a controlled setting. While we were able to provide a first and broad overview of the performance of multiple and diverse measures, more research is needed to enhance the robustness of our findings. Future research should expand the findings to more realistic flight simulations in both civil and military aviation, try to achieve larger sample sizes and also explore the possibilities of using advanced statistical methods, machine learning algorithms or artificial intelligence. This would allow to investigate the extent to which the combination of suitable parameters represents added value beyond the measurement of individual values for the detection of MWL in the aviation context.

## Data Availability

The datasets presented in this study can be found in online repositories. The names of the repository/repositories and accession number(s) can be found below: Data collected by DLR are publicly available here: https://doi.org/10.17605/OSF.IO/YXRKE. Data collected by the German Air Force cannot be shared publicly because of institutional guidelines and restrictions. Data are available upon request from Stefan Sammito (StefanSammito@bundeswehr.org) for researchers who meet the criteria for access to confidential data.
