# Supplementary material for: Examining physiological parameters of mental workload in the cockpit: a multimethod approach
Source: Front Physiol. 2026 Jul 23;17:1893570. doi: 10.3389/fphys.2026.1893570 (PMC13441809; doi:10.3389/fphys.2026.1893570)
Supplement: Supplementary file 1 [file SupplementaryFile1.docx]

**Supplementary Material**

Figure S1. Beta values of HbR per ROI and n-back level. Significant contrasts are marked *p* < .05* and *p* < .01**. Error bars indicate ± 1 *SE*.

Figure S2. Beta values of HbO per ROI and n-back level. Significant contrasts are marked *p* < .05* and *p* < .01**. Error bars indicate ± 1 *SE*.

**Table S1. Significant haemodynamic results for channel-wise t-contrasts in HbR and HbO.**

| Type | Comparison | | Lateralisation | Channel | β (*SE*) | *t* | *p*-value |
| --- | --- | --- | --- | --- | --- | --- | --- |
| HbR | 0 vs. | 1 | left | AF7 – AFF5h | -3.49 (1.05) | -3.34 | .010 |
|  |  |  | right | AFF6h – AF8 | -9.01 (1.12) | -8.07 | < .001 |
|  |  |  |  | FFC4h – FFC6h | 6.21 (1.51) | 4.11 | .001 |
|  |  | 2 | left | AF7 – AFF5h | -5.60 (1.01) | -5.54 | < .001 |
|  |  |  |  | AFF3h – AFF5h | -4.76 (1.69) | -2.82 | .030 |
|  |  |  |  | FCC3h – FFC3h | -7.32 (2.70) | -2.71 | .037 |
|  |  |  | right | FFC4h – FFC2h | -4.91 (1.59) | -3.09 | .018 |
|  |  | 3 | left | AF7 – AFF5h | -8.49 (1.07) | -7.92 | < .001 |
|  |  |  |  | AFF3h – AFF5h | -9.19 (1.78) | -5.16 | < .001 |
|  |  |  |  | FFC5h – AFF5h | -6.08 (2.22) | -2.74 | .036 |
|  |  |  | right | AFF6h – FFC6h | -9.49 (3.68) | -2.58 | .049 |
|  |  |  |  | FFC4h – FFC2h | -4.90 (1.70) | -2.88 | .027 |
|  |  |  |  | FFC4h – AFF4h | -6.55 (2.05) | -3.20 | .014 |
|  | 1 vs. | 2 | left | FFC5h – FFC3h | -4.47 (1.26) | -3.54 | .006 |
|  |  |  | right | AFF6h – AF8 | 9.35 (1.11) | 8.46 | < .001 |
|  |  | 3 | left | AF7 – AFF5h | -5.00 (1.10) | -4.54 | < .001 |
|  |  |  |  | AFF3h – AFF5h | -6.55 (1.80) | -3.63 | .005 |
|  |  |  |  | FFC5h – FFC3h | -5.20 (1.26) | -4.12 | .001 |
|  |  |  | right | AFF6h – AF8 | 7.86 (1.15) | 6.82 | < .001 |
|  |  |  |  | FFC4h – FFC6h | -8.66 (1.56) | -5.55 | < .001 |
|  | 2 vs. | 3 | left | AF7 – AFF5h | -2.89 (1.06) | -2.73 | .036 |
|  |  |  | right | FFC4h – FFC6h | -5.69 (1.54) | -3.69 | .004 |
| HbO | 0 vs. | 1 | left | FFC1h – FFC3h | -25.93 (3.53) | -7.35 | < .001 |
|  |  |  |  | AF7 – AFF5h | -6.15 (2.38) | -2.58 | .049 |
|  |  |  |  | AFF3h – FFC3h | -20.07 (5.06) | -3.97 | .002 |
|  |  |  |  | FFC5h – FFC3h | 7.15 (2.00) | 3.57 | .005 |
|  |  |  | right | AFF6h – AF8 | -28.18 (2.60) | -10.85 | < .001 |
|  |  |  |  | AFF6h – AFF4h | -18.77 (6.25) | -3.00 | .022 |
|  |  |  |  | FFC4h – FFC2h | -16.10 (4.36) | -3.69 | .004 |
|  |  | 2 | left | FFC1h – FFC3h | -29.09 (3.16) | -9.19 | < .001 |
|  |  |  |  | AFF3h – AFp1 | -9.51 (3.13) | -3.04 | .021 |
|  |  |  |  | AFF3h – FFC3h | -24.74 (4.88) | -5.07 | < .001 |
|  |  |  | right | AFF6h – AFF4h | -15.66(4.27) | -3.67 | .004 |
|  |  | 3 | left | FFC1h – FFC3h | -22.97 (3.29) | -6.98 | < .001 |
|  |  |  |  | AFF3h – AFp1 | -14.96 (3.63) | -4.13 | .001 |
|  |  |  |  | AFF3h – FFC3h | -23.27 (5.17) | -4.50 | < .001 |
|  |  |  |  | FFC5h – AFF5h | 14.90 (3.86) | 3.86 | .003 |
|  |  |  | right | AFF6h – AFF4h | -13.15 (4.54) | -2.90 | .027 |
|  | 1 vs. | 2 | left | AFF3h – AFp1 | -14.21 (3.27) | -4.34 | .001 |
|  |  |  |  | AFF3h – FFC5h | -9.68 (3.32) | -2.91 | .026 |
|  |  |  |  | FFC5h – FFC3h | -8.82 (1.87) | -4.72 | < .001 |
|  |  |  | right | AFF6h – AF8 | 32.99 (2.58) | 12.78 | < .001 |
|  |  | 3 | left | AF7 – AFF5h | 7.44 (2.52) | 2.95 | .024 |
|  |  |  |  | AFF3h – AFp1 | -19.65 (3.80) | -5.17 | < .001 |
|  |  |  |  | FFC5h – AFF5h | 12.21 (4.06) | 3.01 | .022 |
|  |  |  |  | FFC5h – FFC3h | -7.82 (1.73) | -4.53 | < .001 |
|  |  |  | right | AFF6h – AF8 | 31.58 (2.66) | 11.86 | < .001 |
|  |  |  |  | AFF6h – AFF4h | 18.22 (6.46) | 2.82 | .030 |
|  | 2 vs. | 3 | left | AF7 – AFF5h | 6.49 (2.41) | 2.70 | .038 |
|  |  |  |  | AFF3h – AFF5h | 10.77 (3.32) | 3.25 | .012 |
|  |  |  |  | FFC5h – AFF5h | 12.66 (3.76) | 3.37 | .009 |
|  |  |  | right | none |  |  |  |

*N* = 14, *df* = 52, *p-*values FDR-corrected.
